# Juvenile idiopathic arthritis in DiGeorge syndrome: a case report and literature review

**DOI:** 10.3389/fimmu.2026.1859919

**Published:** 2026-07-29

**Authors:** Liping Wang, Chenxi Liu, Hua Huang, Xuemei Xu, Xiqiong Han, Shengfang Bao, Zhen Yang, Yanliang Jin, Yingying Jin

**Affiliations:** Department of Rheumatology and Immunology, Shanghai Children’s Medical Center, School of Medicine, Shanghai Jiao Tong University, Shanghai, China

**Keywords:** 22q.11.2 deletion syndrome, DiGeorge syndrome, etanercept, tumor necrosis factor inhibitor, juvenile idiopathic arthritis

## Abstract

**Introduction:**

DiGeorge syndrome (DGS) is an inborn error of immunity characterized by wide phenotypic variability, with a broad spectrum of autoimmune manifestations, including autoimmune cytopenias, thyroiditis, and juvenile idiopathic arthritis (JIA). However, the clinical features of JIA in DGS are not fully understood. Here, we report a case of DGS with JIA and provide a comprehensive review to facilitate early diagnosis and management.

**Case description:**

A 22-month-old girl had a history of frequent respiratory infections and delayed speech after birth. She also had dysmorphic facial features and developmental delay. She had undergone repair of a ventricular septal defect at 3 months of age, during which the thymus was not visualized. Genetic testing revealed a partial heterozygous deletion of 22q11.2. She presented with inflammatory polyarthritis involving bilateral knees and the left hand for 5 months. Antinuclear antibody (ANA) was positive with a titer of 1:320. Anti-cyclic citrullinated peptide antibody showed weakly positive. Magnetic resonance imaging showed synovitis of the affected joints. The patient experienced frequent respiratory infections and delayed speech after birth. Gene examination showed a partial heterozygous deletion of 22q11.2. Thus, she was diagnosed as DGS with JIA. Etanercept was initiated after 4 months of ineffective treatment with naproxen and methotrexate, and remission was observed after 6 months of treatment. Literature review showed a female predominance (62.7%, 32/51) in this population; 80.4% (41/51) were diagnosed before 6 years of age and 62.7% (32/51) had polyarticular involvement. Moreover, 54.2% (26/48) were positive for ANA. Tumor necrosis factor inhibitors (TNFis), mainly etanercept or adalimumab, were used in 20 cases, with more than half of patients responding well to biologics.

**Conclusion:**

The disease has an early onset and polyarthritis is the most common type. Clinicians should suspect underlying DGS in a child presenting with JIA when accompanied by dysmorphic facial features, recurrent infections, congenital heart disease, hypoparathyroidism with hypocalcemia and hyperphosphatemia, and/or decreased T-cell subsets. Treatment with TNFi should be considered when non-steroidal anti-inflammatory drugs and methotrexate are ineffective. Furthermore, follow-up is essential for monitoring adverse drug reactions and proposing prompt intervention.

## Introduction

DiGeorge syndrome (DGS) is an inborn error of immunity associated with a 22q11.2 microdeletion that leads to immunodeficiency. The most common characteristics are cardiovascular disease, facial dysmorphism, hypoparathyroidism, and developmental delay. Patients typically experience frequent infections after birth due to thymus hypoplasia or aplasia. DGS can be divided into partial and complete forms depending on the severity of thymus hypoplasia and T-cell lymphopenia (1, 2). Partial DGS was defined as 22q11.2 deletion with mild to moderate T-cell lymphopenia. The critical regulatory T-cell subpopulation (defective or missing) and/or clones of autoreactive T or B cells are not eliminated during immune development; therefore, this form of DGS often leads to autoimmunity (3–6). These autoimmune features include cytopenias, endocrinopathies, and inflammatory conditions, among which juvenile idiopathic arthritis (JIA) has been increasingly recognized as a significant comorbidity in this population.

JIA is an immune-mediated disease manifested as chronic inflammation of the synovium. It is typically caused by undefined environmental agents overlapping a complex genetic background. Moreover, it is the most common rheumatic disease in children with a prevalence of approximately 1:1,000 in the general population. JIA is observed in patients with DGS. The clinical manifestations of JIA in DGS are mostly recognized based on single-case reports; therefore, its real epidemiologic features and clinical management remain unclear. In this article, we report a case of DGS with JIA, describing the clinical manifestations, treatment, and prognosis. Furthermore, we conducted an overall review of the relevant reports and summarized the currently available knowledge to provide a reference for the diagnosis and management of this disease.

## Case presentation

The patient was a 22-month-old girl born prematurely at 34 weeks and 6 days of gestation, with a birth weight of 2.3 kg. She was diagnosed with congenital heart disease, including a peri-membranous ventricular septal defect (10 mm), a patent foramen ovale (3.2 mm), and a vascular ring (right aortic arch and an aberrant left subclavian artery) at birth. At 3 months of age, she underwent surgical repair of the ventricular septal defect, foramen ovale, vascular ring, and tricuspid valvuloplasty. After birth, she experienced recurrent respiratory tract infections, with two episodes of pneumonia prior to cardiac surgery and two additional episodes after surgical repair. Her physical growth and neurodevelopment were delayed: she achieved independent sitting at 15 months of age, but at 22 months, her height was 78 cm and her weight was 10 kg, yet she was still unable to stand without support, and her expressive language was limited to “Mama” and “Dada”. Based on these milestones, her developmental age was estimated at approximately 12 months, contrasting with her chronological age of 22 months, indicating significant global developmental delay. Physical examination revealed dysmorphic facial features, including micromandible, ocular hypertelorism, and low-set ears. Whole-exome sequencing was performed at a local hospital. All chromosomal coordinates are based on the GRCh37/hg19 assembly. Sequencing data were analyzed by copy number variation analysis. It finally revealed a loss of chr22:18905828-20307511 (1.40 Mb) and chr22:20748918-21414817 (665.90 kb). The genes encompassed by these two intervals—including TBX1, CRKL, DGCR8, PRODH, COMT, SNAP29, and HIRA—are well-established as contributors to the DGS phenotype. To our knowledge, this exact two-fragment deletion configuration has not been previously reported.

She experienced joint symptoms for the first time at the age of 17 months. She developed swelling of both knee joints with pain/restriction of terminal motion. Laboratory evaluation revealed an antinuclear antibody (ANA) titer of 1:320. Magnetic resonance imaging (MRI) demonstrated joint effusion, synovial thickening, and contrast enhancement of both knees. A diagnosis of ANA-positive JIA (oligoarticular) was established. Initial treatment with naproxen (50 mg once daily) and methotrexate (MTX) (6.25 mg once weekly) was administered for 4 months. However, the symptoms of her knees did not improve, and she subsequently developed swelling of her right wrist and left hand at the proximal interphalangeal joint of the fourth finger.

**Figure 1:**
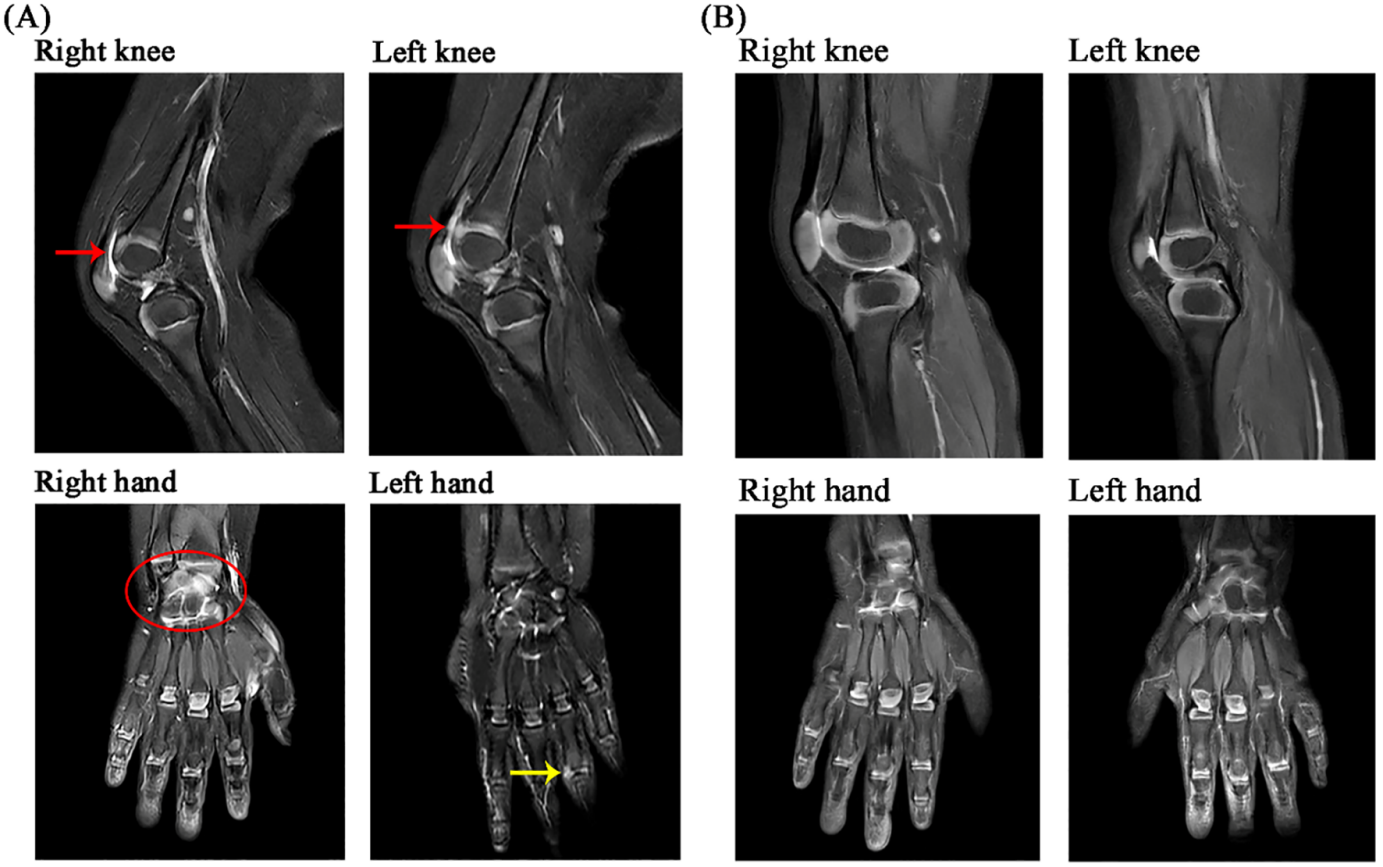
**(A)** MRI findings of the affected joints before TNFi treatment. Synovitis was observed in the biliteral knee (right arrow) and right wrist joints (right circle). Swelling of the soft tissue at the proximal interphalangeal joint of the fourth finger of the left hand, accompanied by tenosynovitis (yellow arrow). **(B)** Synovitis, extension, and swelling of the affected joints were alleviated after 6 months of TNFi treatment. MRI, magnetic resonance imaging; TNFi, tumor necrosis factor inhibitor.

Upon presentation to our hospital at 22 months of age, her knees showed a 25°flexion deformity, resulting from chronic knee arthritis of at least 3 months’ duration, without clinical evidence of active synovitis at the time of examination. Physical examination also confirmed swelling of the left hand. No ocular involvement (uveitis) was detected on ophthalmological examination. The laboratory data from the local hospital 4 months earlier and the repeated tests at our center are shown in **Table 1**, and MRI revealed significant synovitis of the affected joints (**Figure 1A**). Because MTX primarily inhibits T-cell proliferation rather than T-cell development, and her joint symptoms showed insufficient response to NSAIDs and MTX, treatment was switched to etanercept monotherapy. At 6-month follow-up, MRI demonstrated marked remission of synovitis (**Figure 1B**), and the patient was able to walk independently, albeit with an unsteady gait. Specifically, laboratory data including cytokines and inflammatory factor all returned to normal levels after therapy.

**Table 1:** Laboratory characteristics of the patient in a local hospital (4 months earlier) and our institution.

Laboratory characteristics	In the local hospital	In our institution
Antinuclear antibody titers	1:320	1:320
Anti-cyclic citrullinated peptide antibodies	Negative	Weakly positive
Rheumatoid factor	Negative	Negative
CD 3 (No/mm^3^), *n* (reference range)	1,434 (950–1,960)	1,878 (1,400–8,000)
CD 4 (No/mm^3^), *n* (reference range)	927.20 (446–1,100)	1,223 (900–5,500)
CD 8 (No/mm^3^), *n* (reference range)	350.82 (315–808)	482 (400–2,300)
IgA (g/L), *n* (reference range)	0.69+ (0.31–0.67)	0.44 (0.21–1.45)
IgG (g/L), *n* (reference range)	17.20+ (5.09–10.09)	12.8+ (4.7–12.3)
IgM (g/L), *n* (reference range)	0.977− (0.98–1.78)	0.65 (0.41–1.75)
Serum calcium (mmol/L), *n* (reference range)	2.44 (2.10–2.80)	2.58 (2.23–2.80)
Serum phosphate (mmol/L), *n* (reference range)	1.58 (1.37–1.99)	1.88 (1.45–2.10)
PTH (ng/L), *n* (reference range)	ND	10.14 (15.0–65.0)
IL-5 (pg/mL), *n* (reference range)	3.19+ (≤3.1)	5.9 (≤8.7)
Interferon α (pg/mL), *n* (reference range)	4.85 (≤8.5)	4.6 (≤13.2)
IL-2 (pg/mL), *n* (reference range)	9.31+ (≤7.5)	3.7 (≤8.2)
IL-6 (pg/mL), *n* (reference range)	7.32+ (≤5.4)	24.4+ (≤7.0)
IL1β (pg/mL), *n* (reference range)	1.30 (≤12.4)	3.1 (≤12.3)
IL-10 (pg/mL), *n* (reference range)	7.19 (≤12.9)	7.4 (≤9.1)
Interferon γ (pg/mL), *n* (reference range)	9.35 (≤23.1)	7.1 (≤16.2)
IL-8 (pg/mL), *n* (reference range)	23.03+ (≤20.6)	3.6 (≤62.0)
IL-17 (pg/mL), *n* (reference range)	24.79+ (≤21.4)	7.5 (≤19.0)
IL-4 (pg/mL), *n* (reference range)	1.81 (≤8.56)	4.3 (≤11.5)
IL-12P70 (pg/mL), *n* (reference range)	6.87+ (≤3.4)	3.8 (≤8.5)
TNF-α(pg/mL), *n* (reference range)	3.56 (≤16.5)	3.0 (≤8.0)

−, Value is lower than normal ranges; +, Value is higher than normal range; PTH, phytohemaggluttinin; IL, interleukin; TNF, tumor necrosis factor; ND, not determined.

A systematic literature search was conducted in PubMed, Web of Science, and GeenMedical using MeSH terms including “DiGeorge syndrome”, “22q11.2 deletion”, “inborn errors of immunity”, “IgA deficiency”, “juvenile idiopathic arthritis”, “tumor necrosis factor inhibitor”, “etanercept”, “adalimumab”, “biological agents”, and “chronic inflammatory arthritis” combined with the Boolean operators “AND” and “OR”. A total of 10 publications—comprising original articles, case series, case reports, and review—published between 1 January 1997 and 31 December 2025 were identified. The relevant literature and characteristics of chronic inflammatory arthritis (CIA) in 22q11 deletion syndrome are shown in **Table 2** (7–16). Based on our comprehensive review, a total of 51 cases has been reported thus far, including the present case. This disease has an early onset; 80.4% (41/51) were diagnosed before the age of 6 years. There is a predominance of female patients (62.7%, 32/51) in this population. According to the International League of Associations for Rheumatology (ILAR) criteria, the arthritis patterns in these 51 patients were polyarticular (62.7%, 32/51) and oligoarticular (37.3%, 19/51). This condition typically affects both large and small joints interchangeably. Additionally, no cases of axial joint involvement (such as sacroiliitis or spondylitis) have been documented in the literature, nor were there any cases meeting the criteria for systemic or psoriatic JIA to date. In terms of serological findings, ANA positivity was observed in 54.2% (26/48) of cases, RF positivity in 4.7% (2/43), and CCP positivity in 3.8% (1/26). T cells were abnormal in 42.9% (21/49) of patients and immunoglobulin A (IgA) deficiency was detected in 22.0% (11/50) of patients. NSAIDs, MTX, and oral corticosteroids are the primary choice for managing joint symptoms; 46.7% (21/45) were treated with biologics. Tumor necrosis factor inhibitor (TNFi) of etanercept or adalimumab was also used in 20 cases, in which only two cases prescribed TNFi as monotherapy. In addition, three cases (two of them had not response in using TNFi) introduced abatacept as combination therapy. In a follow‑up of 6 months to 20 years following biologic therapy, complete remission was observed in 12 cases, partial remission in 3 cases, and disease relapse in 6 cases.

**Table 2:** Characteristics of chronic inflammatory arthritis (CIA) in 22q11 deletion syndrome (22q11 DS).

Clinical information	Sullivan KE (1997) (7)	Verloes A (1998) (8)	Davies K (2001) (9)	Pelkonen P (2002) (10)	Sato S (2009) (11)	Salehzadeh F (2014) (12)	Sestan M (2019) (13)	Accardo V (2024) (14)	de Oliveira-Sobrinho RP (2024) (15)	Liebling E (2025) (16)
Case number	3	3	5	3	1	1	1	2	1	30
Male/Female	2/1	1/2	1/4	1/2	0/1	1/0	1/0	1/1	1/0	10/20
Age onset of CIA (years)	1.4–5.0	1.0–4.0	1.5–11	0.7–1.4	4.0	5.0	3.0	2.0–6.0	0.7–3.0	3.0
Type of CIA (Poly)	3 cases	3 cases	5 cases	3 cases	1 case	None	1 case	None	1 case	15 cases
Age at 22q11DS diagnosis (years)	0.7–5.0	0.8–7.0	0.4–10.0	0–0.7	6.0	10.0	14.0	2.0–6.0	0.7–3.0	0–15.0
Abnormal T cell	3 cases	None	3 cases	1 case	1 case	NM	1 case	1 case	NM	10 cases
IgA deficiency	2 cases	None	2 cases	1 case	1 case	None	1 case	None	None	4 cases
ANA (+)	2 cases	None	3 cases	None	None	None	None	1 case	None	19 cases
RF/CCP (+)	None	None	2 cases	None	None	NM	NM	None	None	None
Treatment of JIA	NM	MTX, GC	MTX, GC	NM	NSAIDs, MTX	NM	NSAIDs, MTX, GC, TNFi	NSAIDs, MTX, GC, TNFi	NM	NSAIDs MTX, GC, TNFi

Poly, polyarticular; Olig, oligoarthritic; NM, not mentioned; +, positive; ANA, antinuclear antibody; RF, rheumatoid factor; CCP, citrullinated peptide antibodies; NSAIDs, non-steroidal anti-inflammatory drugs; MTX, methotrexate; GC, glucocorticoid.

## Discussion

JIA is an umbrella term for arthritis of unknown origin lasting more than 6 weeks in children. In the general population, JIA is more common in female patients, with a female-to-male ratio of approximately 2:1. Among the ILAR categories, oligoarthritis and polyarthritis are the most prevalent, with incidence rates of 3.7 and 1.4 per 100,000 population, respectively (17). The mean age at diagnosis ranges from 6.5 years for oligoarthritis to 12.2 years for RF-positive polyarthritis (18). However, JIA in patients with DGS is an early-onset condition, with 80.4% of patients diagnosed before 6 years of age. More than half of the patients were ANA-positive, but only three cases tested positive for RF/CCP. Liebling et al. (16) conducted a retrospective analysis of 30 cases with CIA and DGS and concluded that the arthritis in DGS may differ from classical JIA, as CCP and RF antibodies were consistently negative in these patients. Therefore, future studies with larger cohorts are warranted to further define the relationship between JIA and chronic arthritis in patients with DGS.

Defective T-cell function is the hallmark of DGS anomaly. The T-cell lymphopenia represents a hallmark of the underlying 22q11.2 deletion syndrome, which is classically associated with thymic hypoplasia and impaired T-cell output. This defect involves not only the absolute reduction of CD3^+^, CD4^+^, and CD8^+^ subsets but also a more fundamental disturbance in the composition and function of the T-cell compartment. These T-cell aberrations provide a mechanistic link between the immunodeficiency phenotype of 22q11.2DS and the early-onset, polyarticular arthritis observed in our index case, reinforcing the notion that immune dysregulation—rather than mere immunodeficiency—underpins the pathogenesis of JIA in this genetic condition. Nevertheless, immunoglobulin deficiency due to B-cell dysfunction has also been reported. IgA deficiency is the most commonly identified primary antibody deficiency. Patients with IgA deficiency may be asymptomatic; however, some may experience complications, including frequent infections, allergies, and autoimmune diseases (11, 19). In the current analysis, 11 patients had IgA deficiency, and 4 of them experienced recurrent infections. Of note, the present case had normal immunoglobulin levels, which is compatible with partial DGS, in which T- and B-cell counts may be within the normal range.

Both autoimmune and environmental factors contribute to the pathogenesis of JIA. Multiple cytokines, including interleukin (IL)-1β, IL-6, IL-2, and TNF-α, are involved in disease development (20, 21). Measurement of cytokine levels provides important information regarding the molecular mechanisms of synovitis and may guide early targeted therapy. However, the molecular mechanisms underlying JIA in patients with DGS remain largely unknown. Sato et al. (11) reported a case of JIA with IgA deficiency in a patient with DGS. They demonstrated that T cells, B cells, macrophages, and neutrophils can modulate joint inflammation through the regulation of proinflammatory cytokines such as IL-8, IL-6, macrophage inflammatory protein-1β (MIP-1β), and monocyte chemoattractant protein-1 (MCP-1). Furthermore, IL-10 and IL-5 may be associated with the development of synovitis and IgA deficiency. In the present case, laboratory tests also revealed elevated IL-6 levels. However, serum TNF-α level before anti-TNF therapy was within the normal range. Although the serum TNF-α level in our patient was within the normal range before treatment, this does not preclude a good response to etanercept. Indeed, Morozova et al. (22) demonstrated that serum TNF-α levels are not a reliable biomarker of disease activity or therapeutic response in patients with JIA treated with anti-TNF agents. Nevertheless, large-scale cohort studies and animal experiments are warranted to elucidate the association between cytokine profiles and synovitis in patients with DGS with concurrent JIA.

Recent years have seen a paradigm shift in JIA treatment strategies. While NSAIDs and immunosuppressants remain the standard first-line agents, TNFi are recommended to achieve early disease remission. To date, 11 cases of JIA in patients with DGS have been reported to achieve remission following TNFi therapy. Etanercept is a soluble receptor fusion protein that does not fix complement or induce antibody-dependent cell-mediated cytotoxicity, which may be advantageous in patients with underlying immune deficiency. It has a well-established safety and efficacy profile in pediatric polyarticular JIA, with a lower risk of inducing anti-drug antibodies compared to monoclonal antibodies. Previous case reports and small series have successfully used etanercept in patients with JIA with DGS without significant infectious complications. Otherwise, several studies have demonstrated that MTX suppresses T-cell proliferation at clinically relevant concentrations and influences the differentiation of naive T cells into effector and memory cells following antigen stimulation in peripheral T-cell development (23, 24). Therefore, given that the patient in the present case did not respond to MTX, etanercept was initiated as monotherapy in the subsequent treatment to avoid excessive immunosuppression. However, the optimal timing for introducing immunosuppressants and biologic agents in patients with DGS remains controversial, as such therapy may increase susceptibility to infections and trigger autoimmunity. Sestan et al. (13) reported adverse effects of TNFi therapy in a patient with DGS and JIA. Although the patient responded well to TNFi, they developed psoriatic rash and interstitial lung infiltrates after 2 years of adalimumab treatment. In the present case, the child showed significant clinical improvement and has remained stable during the 6-month follow-up duration, with no severe infections observed to date. A possible explanation is that our patient had normal T-cell counts and did not receive concomitant MTX.

To place our findings in a clinical context, we briefly detail the presentation, laboratory workup, and therapeutic decision-making in our index patient. This case reinforces that TNFi can be an effective and safe option after conventional therapy failure. However, in the reviewed literature, the subsequent management of patients who did not respond to TNFi was not explicitly reported, and no alternative therapeutic strategies were detailed for this subgroup. Regarding safety, although no increased incidence of infectious complications was specifically noted in these published cases, close monitoring for infections remains essential given the inherent immunodeficiency of patients with DGS.

While TNFi represent an effective treatment option for patients with JIA who fail to respond to NSAIDs and MTX, their administration may not be feasible in many resource-limited countries due to financial constraints, limited availability, or logistical challenges. In such settings, alternative treatment modalities should be considered. Leflunomide, a conventional synthetic disease-modifying anti-rheumatic drug (DMARD), has been shown to be an effective and safe therapeutic option in patients with polyarticular JIA who are intolerant or non-responsive to MTX (25). More recently, tofacitinib, a Janus kinase (JAK) inhibitor, has demonstrated good efficacy and tolerability in children with refractory polyarticular JIA (26). Given its oral administration and lower cost compared to biologics, tofacitinib may serve as a valuable alternative in low- and middle-income countries. Therefore, while TNFi therapy remains the preferred biologic option after conventional DMARD failure, leflunomide and JAK inhibitors represent viable alternatives in settings where biologics are unavailable, unaffordable, or ineffective, and their roles in the treatment algorithm for DGS with JIA warrant further investigation.

## Conclusion

JIA in patients with DGS is commonly observed and is typically characterized by early onset, before 6 years of age. Polyarthritis is the most frequent subtype in this population. Clinicians should suspect underlying DGS in a child presenting with JIA when accompanied by characteristic dysmorphic facial features, a history of recurrent infections, congenital heart disease, hypoparathyroidism with hypocalcemia and hyperphosphatemia, and/or decreased T-cell subsets, as these features may indicate an underlying 22q11.2 deletion. TNFi therapy should be considered in patients who do not respond adequately to NSAIDs and MTX. Long-term regular follow-up is essential for monitoring potential adverse drug effects and enabling timely intervention.

## Data Availability

The original contributions presented in the study are included in the article/**Supplementary Material**. Further inquiries can be directed to the corresponding authors.

## Supplementary material

Supplementary Table 1 Characteristics of chronic inflammatory arthritis (CIA) in the fifty 22q11 deletion syndrome (22q11 DS) cases. Ref, reference; ANA, antinuclear antibodies; CCP, cyclic citrullinated peptide; DMARDs, disease-modifying anti-rheumatic drugs; F, female; IACI, intra-articular corticosteroid injections; IP, interphalangeal joints; M, male; MCP, metacarpophalangeal joints; NSAIDs, non-steroidal anti-inflammatory drugs; RF, rheumatoid factor; TMJ, temporo-mandibular joint; y, years; NM, Not mentioned; -, Negative; +, Positive; MTX, Methotrexate; Poly, polyarticular; Olig, oligoarthritic; N/M, Not mentioned; GC, glucocorticoid; PR, partial remission. *In Reference 16, a total of 30 patients were reported, with 10 cases presenting T-cell lymphopenia and 4 cases presenting IgA deficiency. However, the table in that study did not specify which individual patients had these abnormalities.

## References

[1] Biggs SE Gilchrist B May KR . Chromosome 22q11.2 deletion (DiGeorge syndrome): immunologic features, diagnosis, and management. Curr Allergy Asthma Rep. (2023) 23:213–22. doi: 10.1007/s11882-023-01071-4 36897497 PMC9999075

[2] Mustillo PJ Sullivan KE Chinn IK Notarangelo LD Haddad E Davies EG . Clinical practice guidelines for the immunological management of chromosome 22q11.2 deletion syndrome and other defects in thymic development. J ClinImmunol. (2023) 43:247–70. doi: 10.1007/s10875-022-01418-y 36648576 PMC9892161

[3] Patel PK Chinga ML Yilmaz M Joychan S Ujhazi B Ellison M . Clinical and treatment history of patients with partial DiGeorge syndrome and autoimmune cytopenia at multiple centers. J ClinImmunol. (2024) 44:42. doi: 10.1007/s10875-023-01607-3 38231436

[4] Tison BE Nicholas SK Abramson SL Hanson IC Paul ME Seeborg FO . Autoimmunity in a cohort of 130 pediatric patients with partial DiGeorge syndrome. J Allergy Clin Immunol. (2011) 128:1115–1117.e1-3. doi: 10.1016/j.jaci.2011.06.043 21835443

[5] Giardino G Radwan N Koletsi P Morrogh DM Adams S Ip W . Clinical and immunological features in a cohort of patients with partial DiGeorge syndrome followed at a single center. Blood. (2019) 133:2586–96. doi: 10.1182/blood.2018885244 31015189

[6] Homans JF Tromp IN Colo D Schlösser TPC Kruyt MC Deeney VFX . Orthopaedic manifestations within the 22q11.2 deletion syndrome: a systematic review. Am J Med Genet A. (2018) 176:2104–20. doi: 10.1002/ajmg.a.38545 29159873

[7] Sullivan KE McDonald-McGinn DM Driscoll DA Zmijewski CM Ellabban AS Reed L . Juvenile rheumatoid arthritis-like polyarthritis in chromosome 22q11.2 deletion syndrome (DiGeorge anomalad/velocardiofacial syndrome/conotruncal anomaly face syndrome). Arthritis Rheum. (1997) 40:430–6. doi: 10.1002/art.1780400307 9082929

[8] Verloes A Curry C Jamar M Herens C O'Lague P Marks J . Juvenile rheumatoid arthritis and del(22q11) syndrome: a non-random association. J Med Genet. (1998) 35:943–7. doi: 10.1136/jmg.35.11.943 9832043 PMC1051489

[9] Davies K Stiehm ER Woo P Murray KJ . Juvenile idiopathic polyarticular arthritis and IgA deficiency in the 22q11 deletion syndrome. J Rheumatol. (2001) 28:2326–34. 11669177

[10] Pelkonen P Lahdenne P Lantto R Honkanen V . Chronic arthritis associated with chromosome deletion 22q11.2 syndrome. J Rheumatol. (2002) 29:2648–50. 12465167

[11] Sato S Kawashima H Suzuki K Nagao R Tsuyuki K Hoshika A . A case of juvenile idiopathic polyarticular arthritis complicated by IgA deficiency in 22q11 deletion syndrome. Rheumatol Int. (2011) 31:1089–92. doi: 10.1007/s00296-009-1245-4 20012054

[12] Salehzadeh F Bagheri A . Association of juvenile idiopathic arthritis and Digeorge syndrome; a case report. Iran J Pediatr. (2014) 24:334–6. PMC4276593 25562032

[13] Sestan M Kifer N Frkovic M Laskarin AM Jelusic M . Adverse effects of TNF inhibitor in a patient with DiGeorge syndrome and juvenile idiopathic arthritis. Ann Allergy Asthma Immunol. (2020) 124:207–8. doi: 10.1016/j.anai.2019.11.006 31734327

[14] Accardo V Pagnini I Maccora I Marrani E Mastrolia MV Simonini G . Safety and efficacy of biologic immunosuppressive treatment in juvenile idiopathic arthritis associated with inborn errors of immunity. Front Pediatr. (2024) 12:1353825. doi: 10.3389/fped.2024.1353825 38468871 PMC10925618

[15] de Oliveira-Sobrinho RP Appenzeller S Holanda IP Heleno JL Jorente J Vieira TP . Genome sequencing in an individual presenting with 22q11.2 deletion syndrome and juvenile idiopathic arthritis. Genes (Basel). (2024) 15:513. doi: 10.3390/genes15040513 38674447 PMC11049871

[16] Liebling E Freychet C Guarnieri V Jelusic M López JA Kallinich T . Chronic inflammatory arthritis in 22q11.2 deletion (DiGeorge) syndrome: a multicentric study. Orphanet J Rare Dis. (2025) 20:603. doi: 10.1186/s13023-025-04088-2 41287064 PMC12642041

[17] Martini A Lovell DJ Albani S Brunner HI Hyrich KL Thompson SD . Juvenile idiopathic arthritis. Nat Rev Dis Primers. (2022) 8:5. doi: 10.1038/s41572-021-00332-8 35087087

[18] Hyrich KL Lal SD Foster HE Thornton J Adib N Baildam E . Disease activity and disability in children with juvenile idiopathic arthritis one year following presentation to paediatric rheumatology. Results from the Childhood Arthritis Prospective Study. Rheumatol (Oxford). (2010) 49:116–22. doi: 10.1093/rheumatology/kep352 19926670 PMC2789587

[19] Finocchi A Di Cesare S Romiti ML Capponi C Rossi P Carsetti R . Humoral immune responses and CD27+ B cells in children with DiGeorge syndrome (22q11.2 deletion syndrome). Pediatr Allergy Immunol. (2006) 17:382–8. doi: 10.1111/j.1399-3038.2006.00409.x 16846458

[20] Gallagher KT Bernstein B . Juvenile rheumatoid arthritis. Curr Opin Rheumatol. (1999) 11:372–6. doi: 10.1097/00002281-199909000-00008 10503657

[21] Tomé C Oliveira-Ramos F Campanilho-Marques R Mourão AF Sousa S Marques C . Children with extended oligoarticular and polyarticular juvenile idiopathic arthritis have alterations in B and T follicular cell subsets in peripheral blood and a cytokine profile sustaining B cell activation. RMD Open. (2023) 9:e002901. doi: 10.1136/rmdopen-2022-002901 37652558 PMC10476142

[22] Morozova N Avramovič MZ Markelj G Toplak N Avčin T . Dynamics of serum levels of TNF-α in a longitudinal follow-up study in 98 patients with juvenile idiopathic arthritis treated with anti-TNF-α biological drugs. Clin Rheumatol. (2024) 43:2287–93. doi: 10.1007/s10067-024-07012-4 38775868 PMC11189329

[23] Cronstein BN Aune TM . Methotrexate and its mechanisms of action in inflammatory arthritis. Nat Rev Rheumatol. (2020) 16:145–54. doi: 10.1038/s41584-020-0373-9 32066940

[24] Nakajima A Hakoda M Yamanaka H Kamatani N Kashiwazaki S . Divergent effects of methotrexate on the clonal growth of T and B lymphocytes and synovial adherent cells from patients with rheumatoid arthritis. Ann Rheum Dis. (1996) 55:237–42. doi: 10.1136/ard.55.4.237 8733440 PMC1010144

[25] Alcântara AC Leite CA Leite AC Sidrim JJ Silva FS Rocha FA . A longterm prospective real-life experience with leflunomide in juvenile idiopathic arthritis. J Rheumatol. (2014) 41:338–44. doi: 10.3899/jrheum.130294 24334641

[26] Iman K Akter L Rahman MM Laila K Islam MI Rahman SA . Treatment of refractory poly articular course juvenile idiopathic arthritis with tofacitinib: extended experience from Bangladesh. PloS One. (2025) 20:e0312174. doi: 10.1371/journal.pone.0312174 39854407 PMC11759992

