# Supplementary material for: Juvenile idiopathic arthritis in DiGeorge syndrome: a case report and literature review
Source: Front Immunol. 2026 Jul 29;17:1859919. doi: 10.3389/fimmu.2026.1859919 (PMC13462456; doi:10.3389/fimmu.2026.1859919)
Supplement: Supplementary file 1 [file Table1.docx]

**Supplementary table 1.** Characteristics of chronic inflammatory arthritis (CIA) in the fifty 22q11 deletion syndrome (22q11 DS) cases

| Ref | Cases | Sex | Age onset of CIA (y) | Type of CIA | Joint involvement | Age at DGS diagnosis (y) | Abnormal T-cell | IgA deficiency | ANA | RF | CCP | Treatment of JIA | Prognosis |
| --- | --- | --- | --- | --- | --- | --- | --- | --- | --- | --- | --- | --- | --- |
| [7] | 1 | F | 5 | Poly | knee and other joints | 5 | Yes | Yes | - | - | - | NM | NM |
|  | 2 | M | 1.58 | Poly | Large and small joints | 0.08 | No | No | **+** | - | - | Naproxen | Remission |
|  | 3 | M | 1.42 | Poly | Knee and other joints | 0.67 | No | Yes | **+** | - | - | Naproxen, GC and MTX | Remission |
| [8] | 4 | F | 4 | Poly | Hips, knees, elbows, hand and wrists | 0.83 | No | No | - | - | - | GC | Joint deformity |
|  | 5 | M | 3 | Poly | Hips, hand wrist and temporomandibular | 2 | No | No | - | - | - | Plaquenil and MTX | NM |
|  | 6 | F | 1 | Poly | Wrists, elbows, ankles, knees, fingers and toes | 7 | No | No | - | - | - | GC | NM |
| [9] | 7 | F | 4 | Poly | Knees, right elbow, hand and feet | 6 | No | No | - | **+** | **+** | IAIC, GC and MTX | Remission |
|  | 8 | F | 11 | Poly | Hips, knees, ankles, hand and feet | 5 | Yes | No | **+** | - | - | NSAID and MTX | Remission |
|  | 9 | M | 3 | Poly | Fingers, wrists, elbows, neck, knees and ankles | 2 | Yes | Yes | **+** | - | - | MTX | Remission |
|  | 10 | F | 1.5 | Poly | Knees and other joints | 21 | NM | Yes | **+** | - | - | NSAID, MTX and GC | Active |
|  | 11 | F | 5 | Poly | Hands and knees | 10 | No | No | - | **+** | - | NSAID and MTX | Remission |
| [10] | 12 | M | 0.67 | Poly | NM |  | No | No | - | - | NM | NM | NM |
|  | 13 | F | 1.08 | Poly | NM |  | Yes | Yes | - | - | NM | NM | NM |
|  | 14 | F | 1.42 | Poly | NM | 0.5 | No | No | - | - | NM | NM | NM |
| [11] | 15 | F | 4 | Poly | Hand and knee | 6 | No | Yes | - | - | NM | Ibuprofen and MTX | Remission |
| [12] | 16 | M | 12 | Poly | Limbs, ankles and right wrist and | 12 | No | No | - | - | NM | NM | NM |
| [13] | 17 | M | 3 | Poly | Large joints | 14 | Yes | Yes | NM | - | NM | NSAIDs, MTX, GC and adalimumab | Remission |
| [14] | 18 | M | 2.25 | Olig | Right ankle | 0.08 | No | No | **+** | NM | NM | NSAIDs, MTX, GC and etanercept | Remission |
|  | 19 | F | 4.16 | Olig | Left knee | 0.17 | Yes | No | - | NM | NM | NSAIDs, MTX and adalimumab | Remission |
| [15] | 20 | M | 3 | Poly | Hands, feet, hip, wrist and knees | NM | NM | NM | - | NM | NM | NM | NM |
| [16]  * | 21 | M | 2 | Olig | Ankle | NM | NM | NM | **+** | NM | NM | NSAIDs, IACI, GC and etanercept | PR |
|  | 22 | M | 3 | Poly | Elbows, hips, knees, and ankles | NM | NM | NM | - | - | - | NSAIDs, DMARDs, IACI, GC, and adalimumab | Active |
|  | 23 | F | 2 | Poly | Knees, wrists, ankle, finger IP, toe dactylitis | NM | NM | NM | **+** | - | NM | DMARDs, IACI, GC, and etanercept | Active |
|  | 24 | F | 9 | Olig | Finger pPeripheral IPs and MCPs, wrist, ankle, TMJ | NM | NM | NM | **+** | - | - | DMARDs | Remission |
|  | 25 | F | 7 | Olig | Knees, ankle, finger dactylitis | NM | NM | NM | - | - | - | DMARDs, IACI, GC, and etanercept | PR |
|  | 26 | M | 11 | Olig | Hips, knees | NM | NM | NM | - | - | - | DMARDs, IACI and GC | Active |
|  | 27 | F | 1 | Olig | Hips, knees | NM | NM | NM | **+** | - | - | NSAIDs, IACI and etanercept | Remission |
|  | 28 | F | 7 | Olig | Hips, knees | NM | NM | NM | **+** | - | - | NSAIDs, GC, and abatacept | Active |
|  | 29 | M | 5 | Poly | Wrists, elbows, shoulder, finger IPs and MCPs, knees, ankles, cervical spine | NM | NM | NM | **+** | - | - | NSAIDs, DMARDs, IACI, GC, and biologicals (etanercept, adalimumab, abatacept) | Remission |
|  | 30 | F | 2 | Poly | Knees, ankles, cervical spine, finger Peripheral IPs | NM | NM | NM | **+** | - | NM | NSAIDs and DMARDs | Remission |
|  | 31 | F | 3 | Poly | Finger IPs and MCPs, wrist, knee, ankles | NM | NM | NM | **+** | - | - | DMARDs, GC, and etanercept | Active |
|  | 32 | F | 3 | Poly | Wrists, elbows, knees, finger MCPs and Peripheral IPs | NM | NM | NM | **+** | - | - | NSAIDs, DMARDs, IACI, GC, and etanercept | Remission |
|  | 33 | F | 3.5 | Olig | Knee, wrists | NM | NM | NM | NM | NM | NM | NSAIDs | Remission |
|  | 34 | M | 12 | Olig | Ankle, knee | NM | NM | NM | **+** | - | NM | DMARDs and etanercept | Remission |
|  | 35 | M | 1.5 | Poly | Elbow, wrists, finger and toe dactily- tis, knees, ankles | NM | NM | NM | - | - | NM | NSAIDs and DMARDs | Active |
|  | 36 | F | 4 | Olig | Knee | NM | NM | NM | **+** | - | - | IACI | Active |
|  | 37 | F | 9 | Olig | Knee | NM | NM | NM | **+** | - | - | IACI and etanercept | Remission |
|  | 38 | F | 6 | Poly | Wrists, knees, ankles | NM | NM | NM | NM | NM | NM | NSAIDs | Active |
|  | 39 | F | 1.5 | Poly | Ankle, knee, wrist, finger Peripheral IPs | NM | NM | NM | **+** | - | - | NSAIDs, DMARDs, IACI and etanercept | Remission |
|  | 40 | M | 2 | Poly | Knees, ankles, cervical spine, fingers Peripheral IPs | NM | NM | NM | **+** | - | NM | NSAIDs | Active |
|  | 41 | M | 1 | Poly | Knees, elbow, wrists, finger MCPs and Perpheral IPs, TMJs | NM | NM | NM | **+** | - | NM | NSAIDs, DMARDs, IACI and GC | PR |
|  | 42 | F | 2 | Olig | Knee, ankle | NM | NM | NM | **+** | - | - | NSAIDs | Remission |
|  | 43 | F | 2 | Poly | Ankles, shoulder, elbows, wrists | NM | NM | NM | - | - | - | NSAIDs | Remission |
|  | 44 | F | 2 | Olig | Knees, ankle | NM | NM | NM | - | - | NM | NSAIDs, DMARDs, IACI, GC, and etanercept | Active |
|  | 45 | F | 1.3 | Poly | Wrist, shoulder, knee, TMJs, finger and toe Peripheral IPs | NM | NM | NM | - | - | NM | NSAIDs, DMARDs, IACI, GC, and etanercept | Remission |
|  | 46 | F | 12 | Olig | Knees | NM | NM | NM | - | - | NM | NSAIDs, DMARDs and IACI | Remission |
|  | 47 | F | 11 | Poly | knee, hip, SI, toe dactylitis | NM | NM | NM | - | - | NM | NSAIDs, DMARDs, IACI and GC | Active |
|  | 48 | M | 2.3 | Poly | knee, elbow, ankles, shoulders | NM | NM | NM | **+** | - | NM | NSAIDs, DMARDs, IACI, GC, and TNFi (etanercept, adalimumab) | Remission |
|  | 49 | M | 2 | Olig | Ankle | NM | NM | NM | **+** | NM | NM | NSAIDs, IACI, GC, and etanercept | PR |
|  | 50 | F | 3 | Olig | Knee | NM | NM | NM | **+** | NM | NM | NSAIDs, DMARDs, IACI, GC, and adalimumab | Active |

Ref: reference; ANA: antinuclear antibodies; CCP: cyclic citrullinated peptide; DMARDs: disease modifying anti-rheumatic drugs; F: female; IACI: intra-articular corticosteroid injections; IP: interphalangeal joints; M: male; MCP: metacarpophalangeal joints; NSAIDs: non-steroidal anti-inflammatory drugs; RF: rheumatoid factor; TMJ: temporo-mandibular joint; y: years; NM: Not mentioned; -: Negtative; +: Positive; MTX: Methotrexate; Poly: polyarticular; Olig: oligoarthritic; N/M: Not mentioned; GC: glucocorticoid; PR: partial remission

*: In Reference 16, a total of 30 patients were reported, with 10 cases presenting T‑cell lymphopenia and 4 cases presenting IgA deficiency. However, the table in that study did not specify which individual patients had these abnormalities.
